# Porous SiO_2_ Nanospheres Modified with ZrO_2_ and Their Use in One-Pot Catalytic Processes to Obtain Value-Added Chemicals from Furfural

**DOI:** 10.1021/acs.iecr.1c02848

**Published:** 2021-11-26

**Authors:** Rocío Maderuelo-Solera, Stefan Richter, Carmen P. Jiménez-Gómez, Cristina García-Sancho, Francisco J. García-Mateos, Juana M. Rosas, Ramón Moreno-Tost, Juan A. Cecilia, Pedro Maireles-Torres

**Affiliations:** † Departamento de Química Inorgánica, Cristalografía y Mineralogía, Facultad de Ciencias, Universidad de Málaga, Campus de Teatinos, Málaga 29071, Spain; ‡ Institute for Organic Chemistry III/Macromolecular Chemistry, Ulm University, Albert Einstein Allee 11, Ulm 89081, Germany; § Departamento de Ingeniería Química, Facultad de Ciencias, Universidad de Málaga, Campus de Teatinos, Málaga 29071, Spain

## Abstract

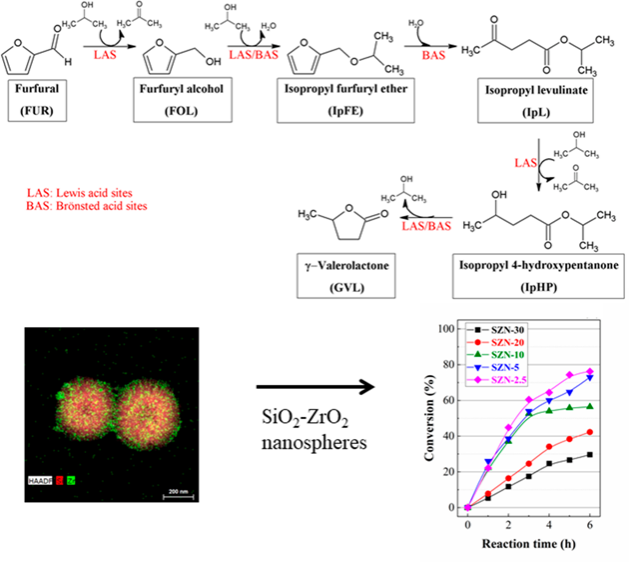

Porous SiO_2_ nanospheres were modified with different loadings of ZrO_2_ to obtain catalysts with a Si/Zr molar ratio from 2.5 to 30. These materials were characterized by X-ray diffraction, transmission and scanning electron microscopies, N_2_ adsorption–desorption at −196 °C, X-ray photoelectron spectroscopy and pyridine and 2–6-dimethylpyridine thermoprogrammed desorption. The characterization of these catalysts has revealed that a high proportion of Zr favors the formation of Lewis acid sites, which are implied in catalytic transfer hydrogenation processes, whereas the low Brönsted acidity promotes a dehydration reaction, being possible to give rise to a large variety of products from furfural through consecutive reactions, such as furfuryl alcohol, i-propyl furfuryl ether, i-propyl levulinate, and γ-valerolactone, in a range of temperature of 110–170 °C and 1–6 h of reaction.

## 1 Introduction

Lignocellulosic biomass has appeared as a highly available feedstock to replace fossil-based resources, being the only sustainable alternative to produce energy, biofuels, and a large spectrum of valuable chemicals. Lignocellulose is mainly composed of cellulose, hemicellulose, and lignin, whose percentages depend on the type of biomass, and yearly environmental or climatic conditions of growing. In the sugar platform, C5 and C6 carbohydrates are converted into value-added chemicals with important applications in many industrial fields.^1^ In this sense, furfural (FUR) is one of the most relevant chemicals derived from lignocellulosic biomass, with an annual production of more than 280 000 tons.^2,3^ FUR is exclusively produced from the dehydration of carbohydrates, mainly pentosans, which are present in agricultural and forestry wastes, using acid catalysts.^4−8^ It was first manufactured by Quaker Oats Company in 1921 from hulls, using diluted sulfuric acid.^9^

This furanic compound possesses interesting applications, since it can be used in the synthesis of resins, as extracting agent of aromatics coming from lubricants, as nematocide, fungicide, or adhesive.^9^ However, the great interest and potential of the FUR molecule is attributed to its chemical structure, with an aldehyde group and a α,β-unsaturated furan ring, which confers it a high reactivity, giving rise to a large variety of chemicals, through hydrogenation, oxidation, condensation, dehydration, and decarbonylation reactions, among others.^5,9^

It has been reported that about 62% of the FUR produced is used for the synthesis of furfuryl alcohol (FOL) by hydrogenation.^10^ The relevance of FOL mainly lies in its wide use in the field of polymers, for foundry resins. In industry, in both gas and liquid phases, FUR hydrogenation is performed with copper chromite as catalyst.^5^ However, this catalyst requires to be replaced by more environmentally friendly Cr-free catalysts.^10^ Most of these catalysts are composed of Cu, Ni, or Pd, for which the catalytic activity, including the nature of products obtained, depends on several factors, such as the hydrogenation capacity of the metal and the nature of the support.^10^

In the past decade, FOL has also been synthesized through catalytic transfer hydrogenation (CTH), with an alcohol, generally secondary, as hydrogen donor to reduce FUR into FOL, without requiring an active metallic phase.^11^ Although the CTH process is relatively novel for the synthesis of FOL, this was discovered nearly 100 years ago by Meerwein, Ponndorf, and Verley.^12−14^ The first studies were carried out through homogeneous catalysis, using some metal complexes and/or alkoxides, which act as Lewis acid sites.^15,16^ Nevertheless, heterogeneous catalysts have progressively appeared as a greener alternative to the traditional homogeneous ones, because they are easily separable and can be reused. Several solid Lewis acid, or basic, catalysts, such as ZrO_2_,^17−22^ Al_2_O_3_,^17,23,24^ Fe_2_O_3_,^25,26^ MgO,^25,27−29^ Zr-,^30−38^ Hf-,^32,38^ or Sn-zeolites^32,35,38,39^ and Zr-,^40−42^ Ru-modified zirconium hydroxide,^43^ Ni- and Ni–W/active carbon,^44^ or Hf- MOFs^45−48^ have been proposed for the CTH of FUR.

**Scheme 1:**
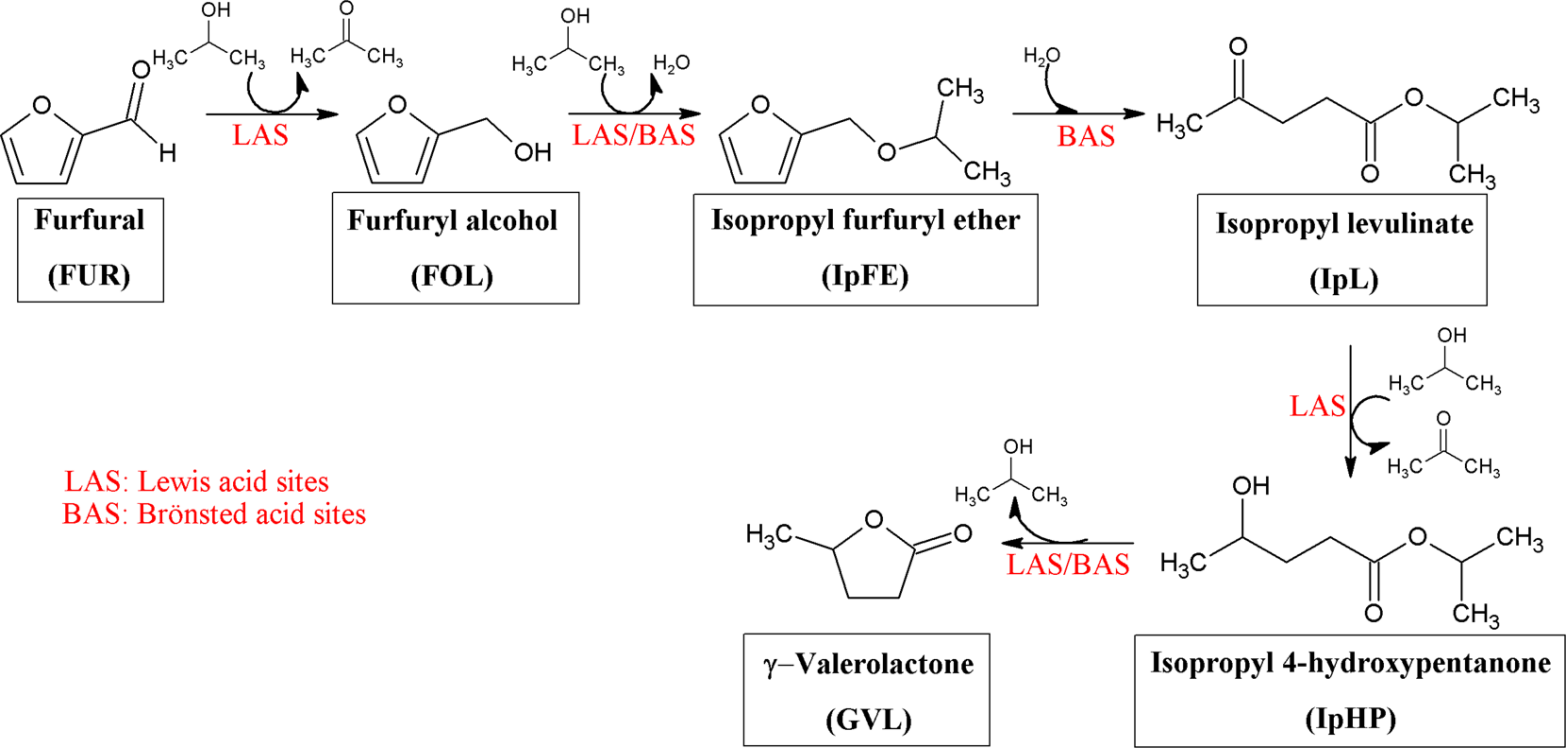
Reaction Scheme of the Consecutive Reactions from FUR to GVL

Nowadays, much research effort is being focused on the design of more efficient bifunctional catalysts, to carry out several catalytic processes in one-pot. However, these one-pot reactions can also have limitations, such as a higher proportion of unwanted products, or difficulty in controlling selectivity, which limit the performance values of the target product. Focusing on FUR, the coexistence of Lewis and Brönsted acid centers has been reported to favor the occurrence of consecutive reactions (Scheme 1), obtaining in all cases high value-added chemicals. Thus, as it was previously indicated, besides the relevance of FOL in the polymer industry, both alkylfurfuryl ether and alkyl levulinate are considered as fuel additives, since both of them increase the cetane index,^49,50^ while γ-valerolactone (GVL) is a relevant platform molecule for the production of biofuels, fuel additives, and polymers.^5^ In previous studies, it has been demonstrated that FUR can be transformed into GVL in one-pot reactions, using Zr- or Hf-zeolites^31−33,35,37,39^ or MOFs.^48^

In the present work, nanoporous spheres of silica, with uniform diameter and narrow pore size distributions, have been synthesized following the methodology proposed by Choi et al.^51^ Their textural and morphological features are appropriate to favor the access and diffusion of reactants and reaction products along the porous network. To provide Lewis acidity, this porous silica was modified by incorporating Zr into the siliceous structure. Then, these Zr-doped porous silica spheres were tested in the valorization of FUR to obtain high value-added chemicals, extending from FOL to GVL. This one-pot process has been generally carried out by using Zr-modified zeolites,^31−33^ while Zr-doped silica has only been employed in the reduction of FUR to FOL.^20,52^

## 2 Materials and Methods

### 2.1 Reagents

The synthesis of Zr-doped silica nanospheres was carried out using tetraethylorthosilicate (99%, Sigma-Aldrich), zirconium propoxide (70% in 1-propanol, Sigma-Aldrich) and zirconium oxychloride octahydrated (99%, Sigma-Aldrich). Cetylpyridinium bromide hydrate (98%, Sigma-Aldrich), cyclohexane (99%, Sigma-Aldrich), and 1-pentanol (99%, Sigma-Aldrich) were also utilized. Urea and hydrochloric acid were purchased from VWR.

Gases used in the characterization and catalytic processes were He (Air Liquide 99.99%), H_2_ (Air Liquide 99.999%), N_2_ (Air Liquide 99.9999%), and N_2_/O_2_ (80/20 vol %).

### 2.2 Synthesis of the Nanospheres

Porous Zr-doped silica nanospheres were prepared following the methodology proposed by Choi et al., with some modifications.^51^ In a typical synthesis, 1 g of cetylpyridinium bromide hydrate was dissolved in 30 mL of water, together with 0.6 g of urea. Then, 12 mmol of silicon and zirconium alkoxides, to obtain a Si/Zr molar ratio between 2.5 and 30, were mixed with 30 mL of cyclohexane and 1.5 mL of 1-pentanol, and added to the first solution, maintaining the stirring for 30 min at room temperature. The resulting solution, after transfer to a Teflon-lined autoclave, was hydrothermally treated under continuous stirring at 120 °C for 6 h.

In an additional synthesis, the Zr species were incorporated in a second step. In this process, 0.6 g of urea and 1 g of cetylpyridinium bromide hydrate were dissolved in 30 mL of water. Then, 2.5 g of tetraethylorthosilicate, 30 mL of cyclohexane and 1.5 mL of 1-pentanol were mixed and added to the first solution, and the resulting solution was stirred at room temperature for 30 min. Later, this was also transferred to a Teflon-lined autoclave and hydrothermally treated, under continuous stirring, at 120 °C for 2.5 h. After cooling to room temperature, the pH of the mother liquor was adjusted to pH 5 by adding a 2 M HCl aqueous solution. Then, 2 mL of a solution of ZrOCl_2_·8H_2_O was added to reach a Si/Zr molar ratio of 5. Subsequently, the obtained mixture was hydrothermally treated again at 120 °C for another 4 h.

In all cases, the resulting nanospheres were obtained by centrifugation, washed with a solution of acetone and water, and then dried at room temperature for 24 h. Finally, the dried materials were calcined in air at 550 °C for 6 h.

The samples were labeled as SZN-*x*, where *x* indicates the Si/Zr molar ratio. In the case of SZN-5-O, the term -O indicates that this catalyst was synthesized in two steps, using zirconium oxychloride as precursor.

### 2.3 Physicochemical Characterization

Powder X-ray diffraction was used to study the crystallinity of SZN-*x* catalysts, with a PANalytical X’Pert PRO diffractometer, with a germanium monochromator and Cu Kα (1.5406 Å) radiation.

The catalyst morphology was evaluated by TEM-EDS, with a FEI Talos F200X, which combines a high-resolution STEM, TEM imaging, and an energy dispersive X-ray spectroscopy (EDS) signal detection. The 3D chemical characterization was obtained from the compositional mapping. The samples were dispersed in ethanol and a drop of the suspension was put on a Formvar/carbon supported Cu grid (300 mesh).

Textural parameters were analyzed by adsorption–desorption of N_2_ at −196 °C, using an automatic ASAP 2020 Micromeritics. Samples were previously outgassed overnight at 150 °C and 10^–4^ mbar. The de Boer’s t-plot method was used to obtain the micropore surface areas,^53^ whereas the specific surface area was deduced from the Brunauer–Emmett–Teller equation (BET), considering a N_2_ cross section of 16.2 Å^2^.^54^ The Nonlocal Density Functional Theory (NLDFT) was applied to determine the pore size distribution from the desorption branch of the isotherm.^55^ The total pore volume was deduced from N_2_ adsorbed at *P*/*P*_0_ = 0.996.

A Physical Electronics PHI5700 spectrometer, with nonmonochromatic Mg Kα radiation (300 W, 15 kV, and 1253.6 eV) and a multichannel detector, was employed to obtain the X-ray photoelectron spectra. A constant pass energy mode at 29.35 eV, with a 720 μm diameter analysis area, was used for recording the spectra. Acquisition and data analysis were performed with a PHI ACCESS ESCA-V6.0F software package, whereas charge referencing was measured against adventitious carbon (C 1s at 284.8 eV). A Shirley-type background was subtracted from the signals, and the fitting of recorded spectra was carried out with Gaussian–Lorentzian curves, for a better determination of different binding energies.

The acidity of catalysts was evaluated by adsorption–desorption of pyridine (Py) and 2,6-dimethylpyridine (DMPy) at 100 °C. These experiments were performed in a thermogravimetric system (CI Electronics) by using 25 mg of dry catalyst. Before pyridine or 2,6-dimethylpyridine adsorption started, samples were outgassed at 150 °C for 2 h. For the adsorption experiments, an inlet partial pressure of Py or DMPy of 0.02 atm was used. This partial pressure was established by saturating 150 cm^3^ (STP)/min of N_2_ with the corresponding organic base in a saturator, at a determined temperature. Once the sample was saturated with Py or DMPy, desorption was carried out at 100 °C, by using a N_2_ flow of 150 cm^3^ (STP)/min. The total amount of Py or DMPy chemisorbed was calculated from the final weight of the sample.

The evaluation of the catalyst leaching was carried out by ICP-MS on a PerkinElmer spectrophotometer (NexION 300D), after digestion of samples with HNO_3_, HCl, and HF in an Anton Paar device (Multiwave 3000).

### 2.4 Catalytic tests

The catalytic tests were performed in glass pressure reactors, with thread bushing (Ace, 15 mL, pressure rated to 10 bar). In a typical experiment, 0.1 mmol of FUR were dissolved in 2-propanol (2-propanol/FUR molar ratio of 50:1), and 100 mg of catalyst was added. Reactors were always purged with helium before the reaction. Reaction time was varied from 1 to 6 h, under continuous stirring (400 rpm), at temperatures between 110 and 170 °C, which was controlled by a thermocouple directly in contact with an aluminum block. After finishing the reaction, the reactor was moved away from the aluminum block and cooled in a water bath. Samples were microfiltered and analyzed by gas chromatography (Shimadzu GC-14A), using a flame ionization detector and a CP-Wax 52 CB capillary column. The calculation of furfural conversion and yield values was carried as follows:
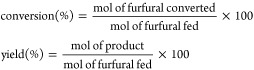


## 3 Results and Discussion

### 3.1 Characterization of Catalysts

**Figure 1:**
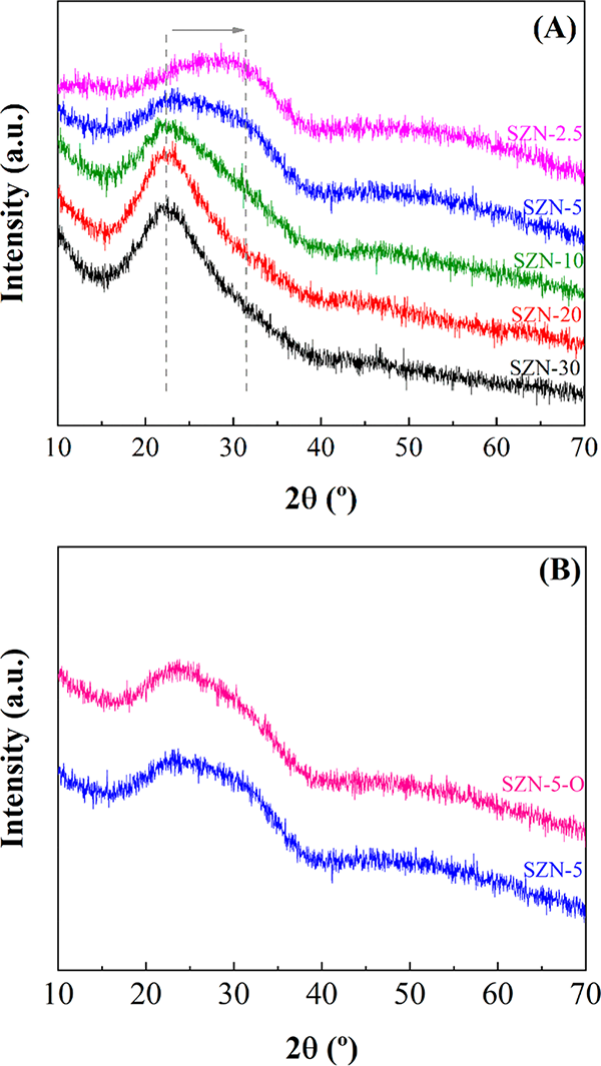
X-ray diffraction patterns of (A) SZN-*x* catalysts with different Si/Zr molar ratio and (B) catalysts with a Si/Zr molar ratio of 5, synthesized by different methodologies.

The crystallinity of SZN-*x* catalysts was studied by X-ray diffraction (Figure 1A). XRD patterns do not show defined peaks, so the segregation of crystalline SiO_2_ or ZrO_2_ must be ruled out. The sample with the lowest Zr content (SZN-30) displays a broad diffraction peak with a maximum about 2θ = 22.4°, which is ascribed to amorphous SiO_2_ forming the walls of nanospheres.^56^ The progressive incorporation of Zr species in the SZN structure provokes a broadening of the diffraction signal and a shift to higher 2θ values, with a maximum about 2θ = 30°, because of the formation of tetragonal zirconia with low crystallinity.^57^ On the other hand, the modification of the Zr source (zirconium oxychloride instead of zirconium propoxide) hardly leads to visible differences in its XRD pattern (Figure 1B).

**Figure 2:**
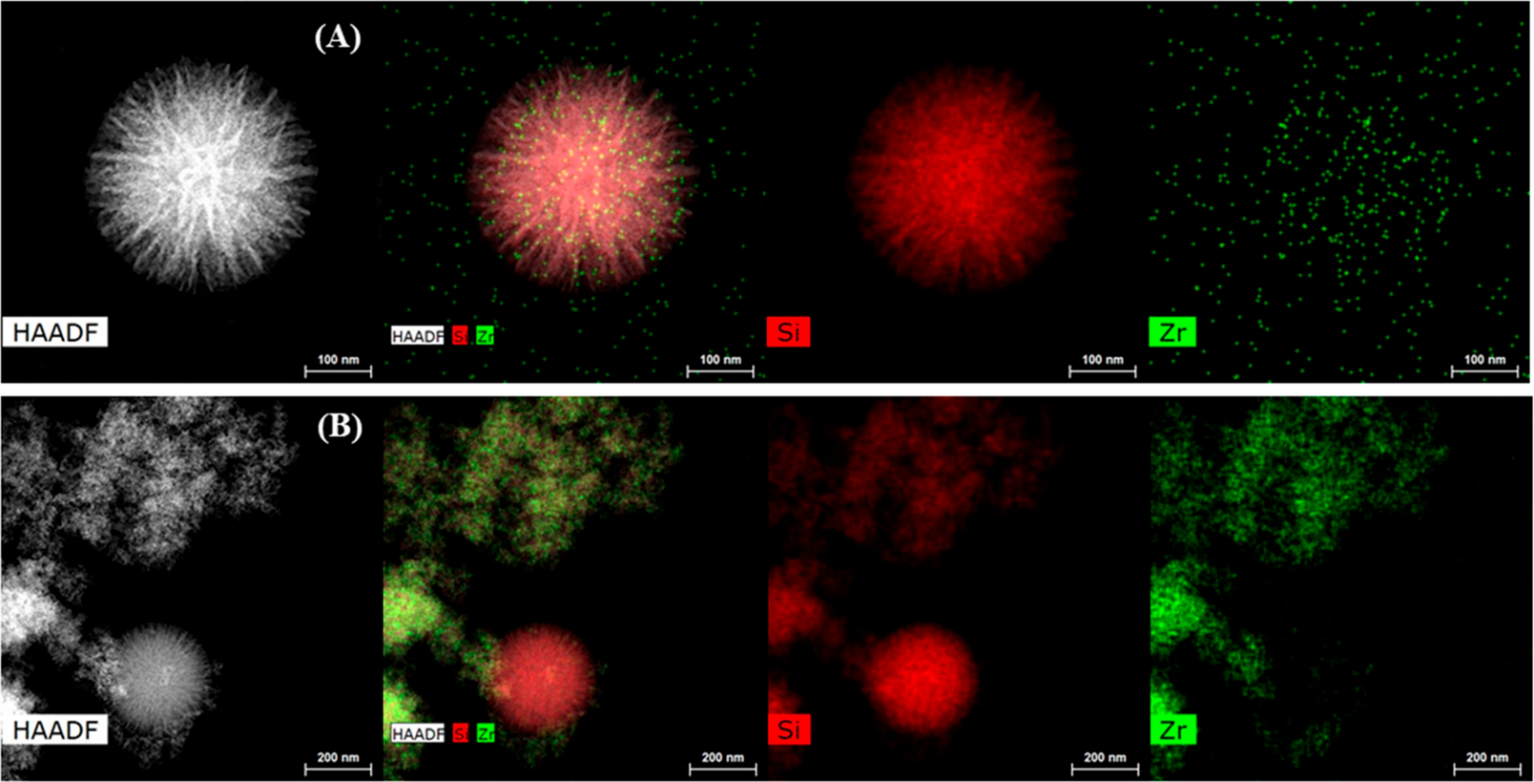
TEM micrographs of (A) SZN-20 and (B) SZN-5. Scale: 100 and 200 nm.

The morphology of the SZN-*x* catalysts was studied by transmission electron microscopy (TEM) (Figure 2). The micrographs show spherical nanoparticles, with diameters between 0.2 and 0.3 μm. The incorporation of Zr species, in the form of alkoxide, provokes a progressive loss of the spherical morphology due to the partial segregation of Zr and Si species in the synthesis step, being more pronounced in the catalyst with the highest Zr loading (SZN-2.5). However, in spite of this, crystalline ZrO_2_ was not detected by XRD (Figure 1), so ZrO_2_ particles must exhibit a low crystallinity.

**Figure 3:**
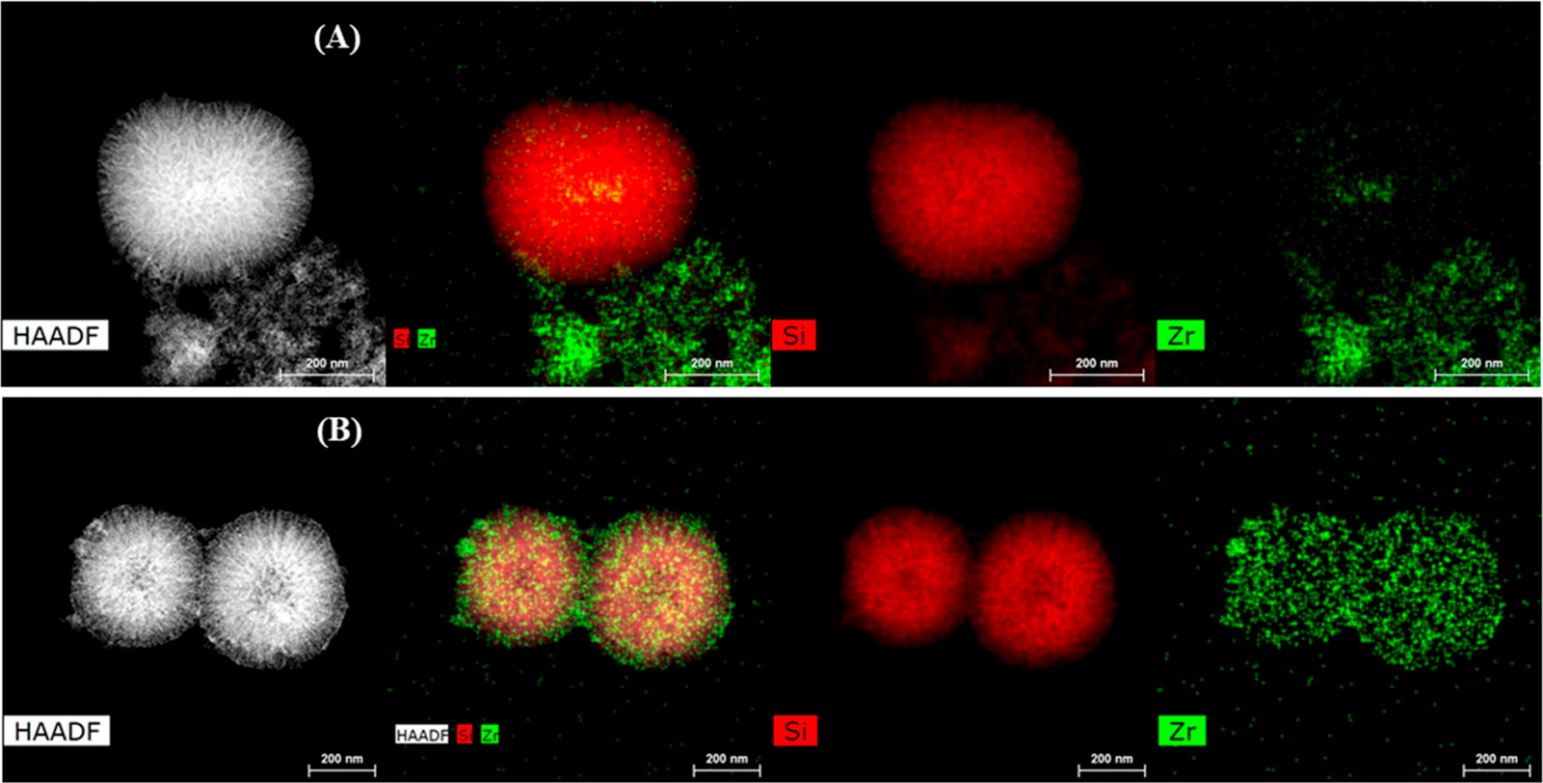
TEM micrographs of (A) SZN-5 and (B) SZN-5-O. Scale: 200 nm.

The comparison between the SZN-*x* catalysts, where Zr species have been incorporated as alkoxide in the synthesis step, or by postsynthesis, using oxychloride, reveals that the simultaneous incorporation of Si and Zr alkoxides gives rise to a higher segregation (SZN-5), whereas the postsynthesis incorporation (SZN-5-O) favors the presence of Zr particles on the external surface of the SZN spheres (Figure 3).

**Figure 4:**
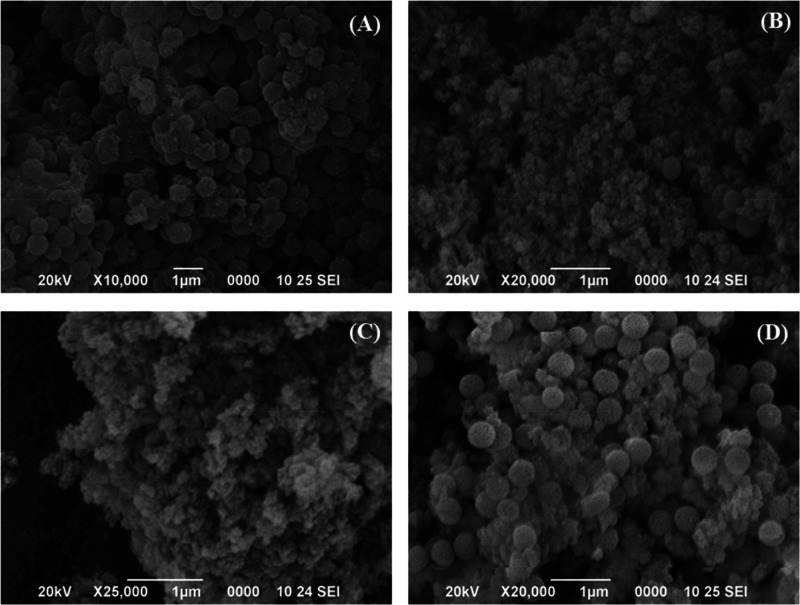
SEM images of (A) SZN-30, (B) SZN-2.5, (C) SZN-5, and (D) SZN-5-O. Scale: 1 μm.

To complete the morphological analysis, a SEM study of the SZN-*x* catalysts was also carried out (Figure 4). These images confirm that the catalyst with the lowest Zr content (SZN-30) maintains the spherical morphology (Figure 4A), which is less visible for higher Zr contents, SZN-2.5 being the most disordered catalyst (Figure 4B), which agrees with the micrographs obtained by TEM (Figures 2 and 3). The comparison between SZN-5 and SZN-5-O catalysts (Figure 4C,D) also confirms that the addition of Zr species in a second step (SZN-5-O) helps to keep the spherical shape of the nanoparticles.

**Figure 5:**
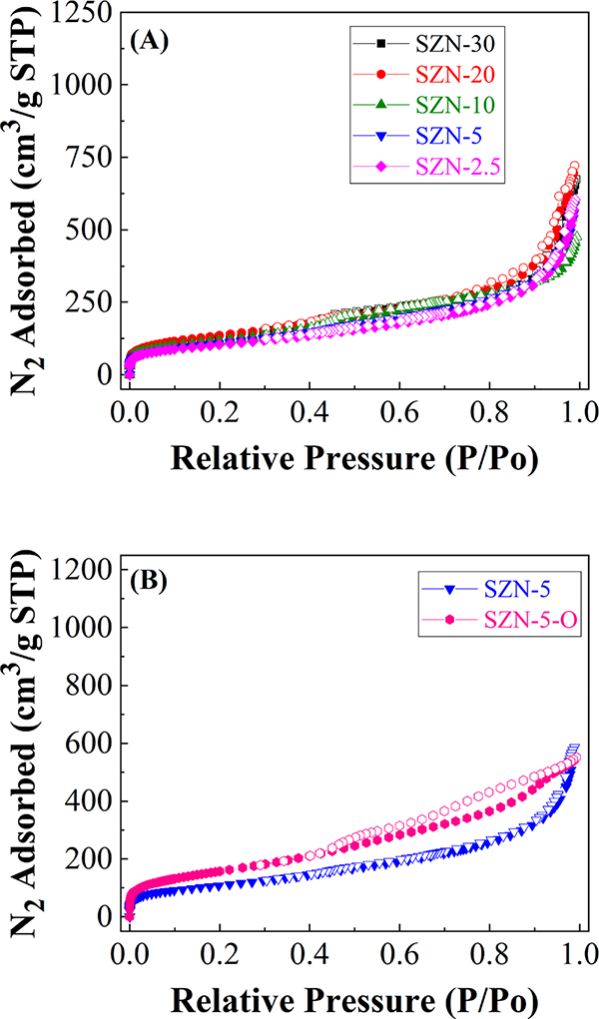
N_2_ adsorption–desorption isotherms of (A) SZN-*x* catalysts with different Si/Zr molar ratio and (B) catalysts with a Si/Zr molar ratio of 5, synthesized by different methodology.

N_2_ adsorption–desorption at −196 °C was employed for the determination of textural properties of the SZN-*x* catalysts (Figure 5). According to the IUPAC classification, isotherms of SZN-*x* catalysts can be classified as Type II, characteristic of macroporous adsorbents, in which the large amount of N_2_ adsorbed at low relative pressures would indicate the existence of micropores or small mesoporores.^55^ On the other hand, the rise of N_2_ adsorbed at high relative pressure suggests that these SZN-*x* samples also possess macropores, which can be ascribed to the voids between adjacent nanospheres, as was previously observed by TEM and SEM (Figures 2–4). In all cases, the hysteresis loop is almost negligible, which would involve that the pore diameter must be close to 5–6 nm, since below this value the hysteresis cycles cannot be observed. The specific surface area, estimated from the BET equation (Table 1),^53^ decreases with the Zr content. However, all SZN-*x* catalysts display *S*_BET_ higher than 375 m^2^ g^–1^. The use of zirconium oxychloride (SZN-5-O) slightly improves the textural properties in comparison to the use of the SZN-5 catalyst, with a similar Si/Zr molar ratio. In addition, the N_2_ adsorption–desorption profile reveals that the SZN-5-O sample adsorbs a lower amount of N_2_ at higher relative pressure, in such a way that this sample must have a lower proportion of voids between nanoparticles.

**Table 1:** Textural properties of SZN-*x* catalysts

catalyst	*S*_BET_ (m^2^ g^–1^)	*t*-plot (m^2^ g^–1^)	*V*_P_ (cm^3^ g^–1^)	*V*_MP_ (cm^3^ g^–1^)
SZN-30	464	27	0.668	0.033
SZN-20	485	13	1.093	0.022
SZN-10	423	17	0.538	0.021
SZN-5	393	14	0.586	0.007
SZN-2.5	376	10	0.591	0.002
SZN-5-O	574	12	0.766	0.003

**Figure 6:**
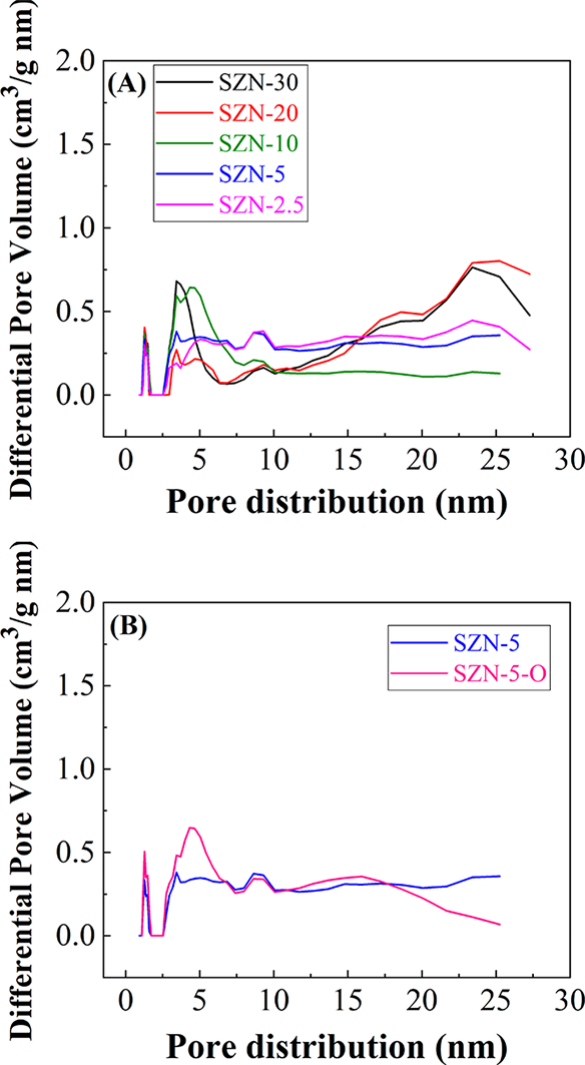
Pore size distribution estimated by DFT of (A) SZN-*x* catalysts with different Si/Zr molar ratio and (B) catalysts with a Si/Zr molar ratio of 5 synthesized by different methodology.

The pore size distribution of the SZN-*x* catalysts was determined by DFT calculations (Figure 6).^54^ All samples display a first maximum about 1.35 nm, confirming the microporosity of samples, as well as a broad band between 2 and 8 nm. Moreover, the samples evidence a slight macroporosity, associated with interparticle voids.

The analysis of the acidity of the SZN-*x* catalysts was carried out by the adsorption of pyridine and 2,6-dimethylpyridine. Many studies have estimated the total acidity by NH_3_ temperature-programmed desorption. Considering that NH_3_ molecules are very small, they can access acidic sites, where bulkier molecules, such as FUR, could be hampered. However, the dimensions of furfural, pyridine, and 2,6-dimethylpyridine are more similar, so the acidity values can be more adjusted to reality. The total acidity, determined by pyridine adsorption (Table 2),^27,58^ reveals that the concentration of acid sites is straightly related to the Zr loading, increasing from 85 to 394 μmol g^–1^ for SZN-30 and SZN-5 catalysts, respectively. It is striking that the incorporation of a higher Zr content in SZN-2.5 decreases the amount of total acid sites. This could be related to the textural properties, since the SZN-2.5 catalyst showed poorer textural properties than that observed for the SZN-5 catalyst (Table 1). In regard to the SZN-5 and SZN-5-O catalysts, they show close acidity values. In spite of SZN-5-O catalyst displaying both a higher specific surface area and higher proportion of Zr species on the catalyst surface, the simultaneous incorporation of Si and Zr in the form of alkoxides (SZN-5) provides a greater acidity.

**Table 2:** Pyridine and 2,6-Dimethylpyridine Adsorption of SZN-*x* Catalysts

catalyst	Pyr ads (μmol g^–1^)	2,6-DMPyr ads (μmol g^–1^)	Pyr ads −2,6-DMPyr ads (μmol g^–1^)
SZN-30	85	45	40
SZN-20	140	44	96
SZN-10	302	96	206
SZN-5	394	80	314
SZN-2.5	346	97	250
SZN-5-O	385	34	341

By using temperature-programmed desorption of 2,6-dimethylpyridine, it is feasible to selectively quantify the amount of Brönsted acid sites (Table 2).^59^ The contribution of Brönsted acidity is relatively low in comparison to the total acidity, as determined from pyridine adsorption, so most of the acid sites should be attributed to the existence of Lewis acid sites, which are ascribed to unsaturated Zr species.^60^ These data agree with previous works, where Zr species mainly provided Lewis acidity, while Brönsted acid sites could be associated with silanol groups. The Brönsted acidity can be affected by the calcination of organic matter at 550 °C, which is an exothermic process, where most of silanol groups could be dehydroxylated to siloxane groups, decreasing the concentration of Brönsted acid sites.^61^

**Figure 7:**
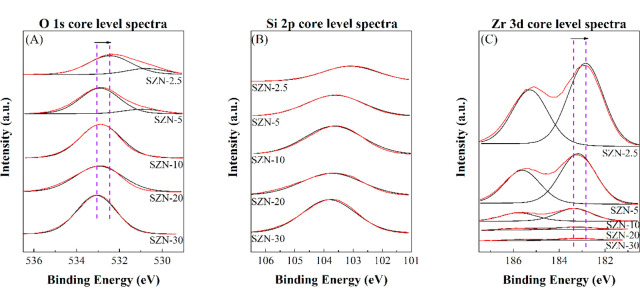
(A) O 1s, (B) Si 2p, and (C) Zr 3d core level spectra of SZN-*x* catalysts with different Si/Zr molar ratio.

**Figure 8:**
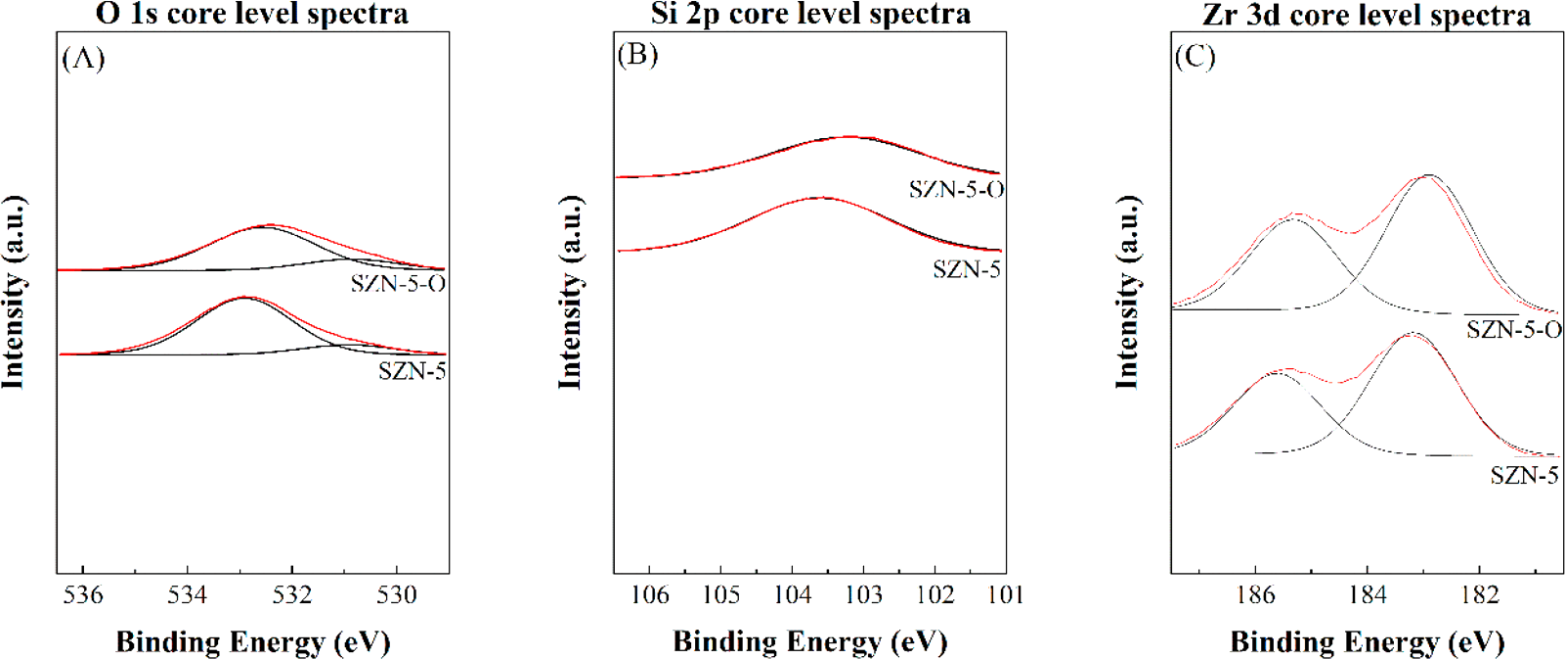
(A) O 1s, (B) Si 2p, and (C) Zr 3d core level spectra of SZN-5 synthesized by different methodologies.

The surface chemical composition of the SZN-*x* catalysts, including the oxidation state of chemical species, has been determined by XPS (Figures 7 and 8 and Table 3). The analysis of the O 1s core level spectrum of the sample with the lowest Zr content (SZN-30) (Figure 7A) shows the presence of a single contribution located about 533.0 eV, which is typical of Si–O–Si bonds.^62^ The incorporation of a higher amount of Zr provokes a shift of this contribution to lowest binding energy values, in such a way that the SZN-2.5 catalyst displays a signal about 532.5 eV, since the electronic density of O atoms is modified in Si–O–Zr bonds. In addition, it is worth noting the appearance, for the catalyst with the highest Zr content (SZN-2.5), of a new contribution at 530.5 eV, which can be assigned to the presence of well-dispersed ZrO_2_ (Zr–O–Zr) on its surface.^62^ The comparison of the SZN-*x* catalyst synthesized with different methods (SZN-5 and SZN-5-O) (Figure 8A) reveals that the O 1s core level spectra display both contributions described previously with similar intensity ratio. However, the main band of the SZN-5-O catalyst is slightly shifted to lower binding energy values, which would indicate that a higher proportion of Zr species could change the electronic density in this catalyst in comparison to SZN-5.

The study of the Zr 3d core level spectra also shows a shift of the binding energy at lower values with the Zr loading, reaching a value of 182.8 eV for SZN-2.5 (Figure 7C). However, this value is higher than that of pure ZrO_2_ (182.2 eV).^62^ Therefore, the incorporation of Zr species in the siliceous framework, at least partially, can be inferred. The higher binding energy of the SZN-*x* catalysts would also reveal a higher ionic nature in the Zr–O–Si bonds compared to Zr–O–Zr, which could create stronger Lewis acid sites in these catalysts.^63,64^

The analysis of the surface chemical composition of the SZN-*x* catalysts (Table 3) reveals that the surface Si/Zr molar ratio is much higher than expected when a low Zr content is incorporated. These data could suggest a faster hydrolysis of Zr alkoxide in comparison to Si alkoxide, causing a proportion of the Zr species to be trapped in the walls of porous silica.^52^ As the incorporated Zr increases, the Si/Zr molar ratio resembles the theoretical values. In fact, the catalyst with the highest Zr content (SZN-2.5) presents lower values than the theoretical ones, a greater amount of Zr being on the surface than expected, confirming that Zr and Si alkoxides possess different hydrolysis rates. In this sense, TEM micrographs revealed a partial segregation of the Zr species, mainly for those catalysts with a higher Zr content. This should cause an increase in the Zr concentration on the catalyst surface.

**Table 3:** XPS Data of SZN-*x* Catalysts

	atomic concentration, %/(binding energy, eV)				
catalyst	C 1s	O 1s	Si 2p	Zr 3d	Si/Zr atomic ratio
SZN-30	3.21/(284.8)	66.99/(532.9)	28.75/(103.6)	0.11/(183.0)	261.36
	0.66/(286.4)				
	0.07/(289.4)				
SZN-20	3.51/(284.8)	66.89/(532.9)	28.29/(103.7)	0.16/(182.9)	176.81
	1.04/(286.4)				
	0.13/(289.4)				
SZN-10	4.37/(284.6)	66.41/(532.7)	27.24/(103.3)	0.98/(182.8)	27.79
	0.78/(286.4)				
	0.22/(289.4)				
SZN-5	3.49/(284.8)	59.39/(532.7)	23.70/(103.2)	4.33/(183.0)	5.47
	0.63/(286.5)	7.85/(530.5)			
	0.21/(289.4)				
SZN-2.5	3.73/(284.8)	67.50	19.89/(103.0)	8.46/(182.8)	2.35
	0.54/(286.7)	53.34/(532.3)			
	0.10/(289.6)	14.15/(530.5)			
SZN-5-O	3.88/(284.8)	58.66/(532.4)	21.04/(103.1)	5.56/(182.9)	3.78
	0.64/(286.6)	5.08/(530.5)			
	0.16				

The comparison of the surface chemical composition between SZN-5 and SZN-5-O catalysts (Table 3) reveals that the addition of the Zr species after the formation of silica nanospheres leads to a lower Si/Zr molar ratio (3.78 versus 5.47) due to the presence of Zr species on the surface of silica nanospheres.

### 3.2 Catalytic Tests

**Figure 9:**
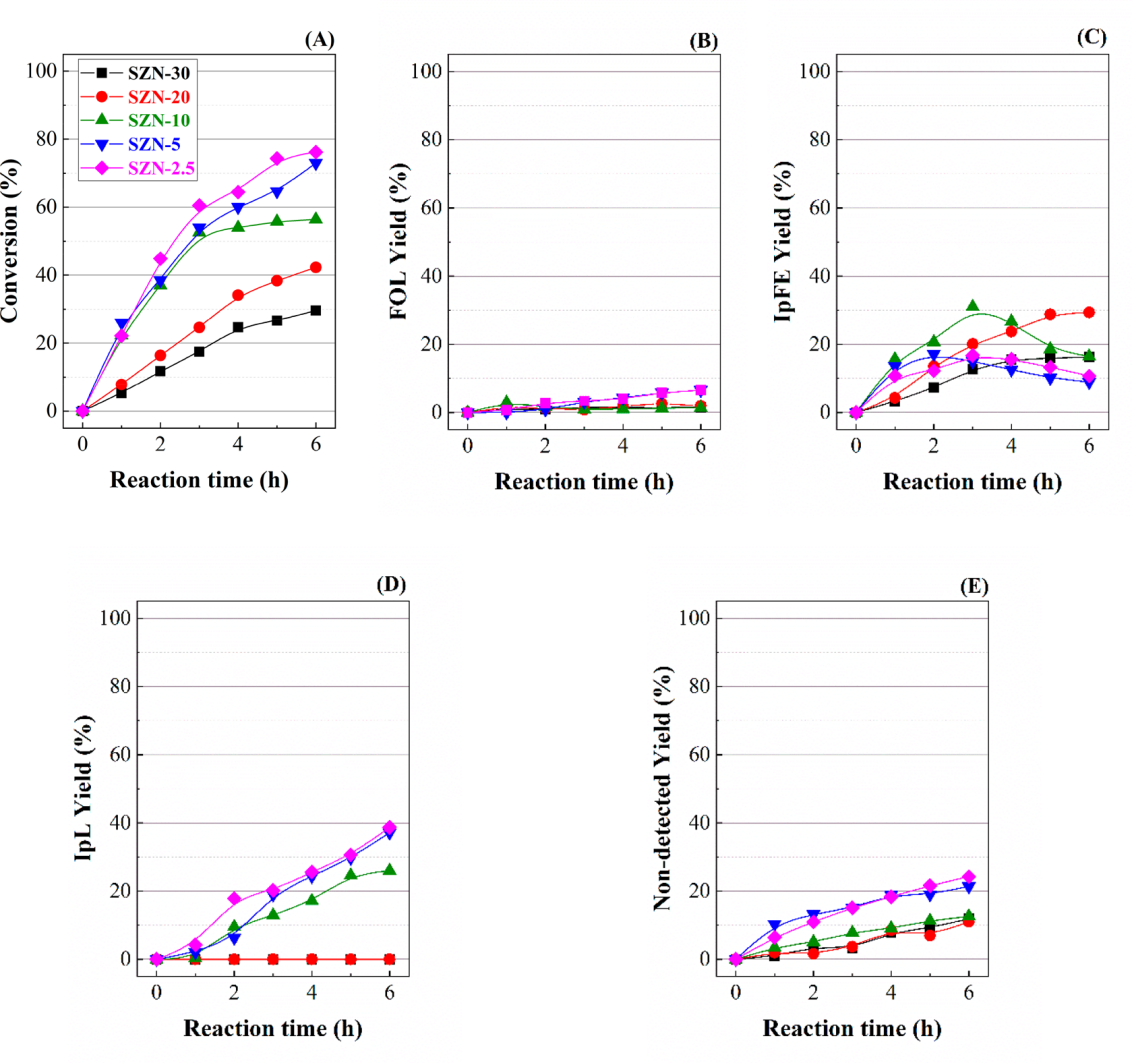
(A) FUR conversion, (B) FOL yield, (C) IpFE yield, (D) IpL yield, and (E) non-detected product yield, in the CTH reaction of FUR using SZN-*x* catalysts with different Si/Zr molar ratios (experimental conditions: temperature, 110 °C; i-Pr–OH/FUR molar ratiom 50; 0.1 g of catalyst; FUR/catalyst weight ratio, 1).

**Figure 10:**
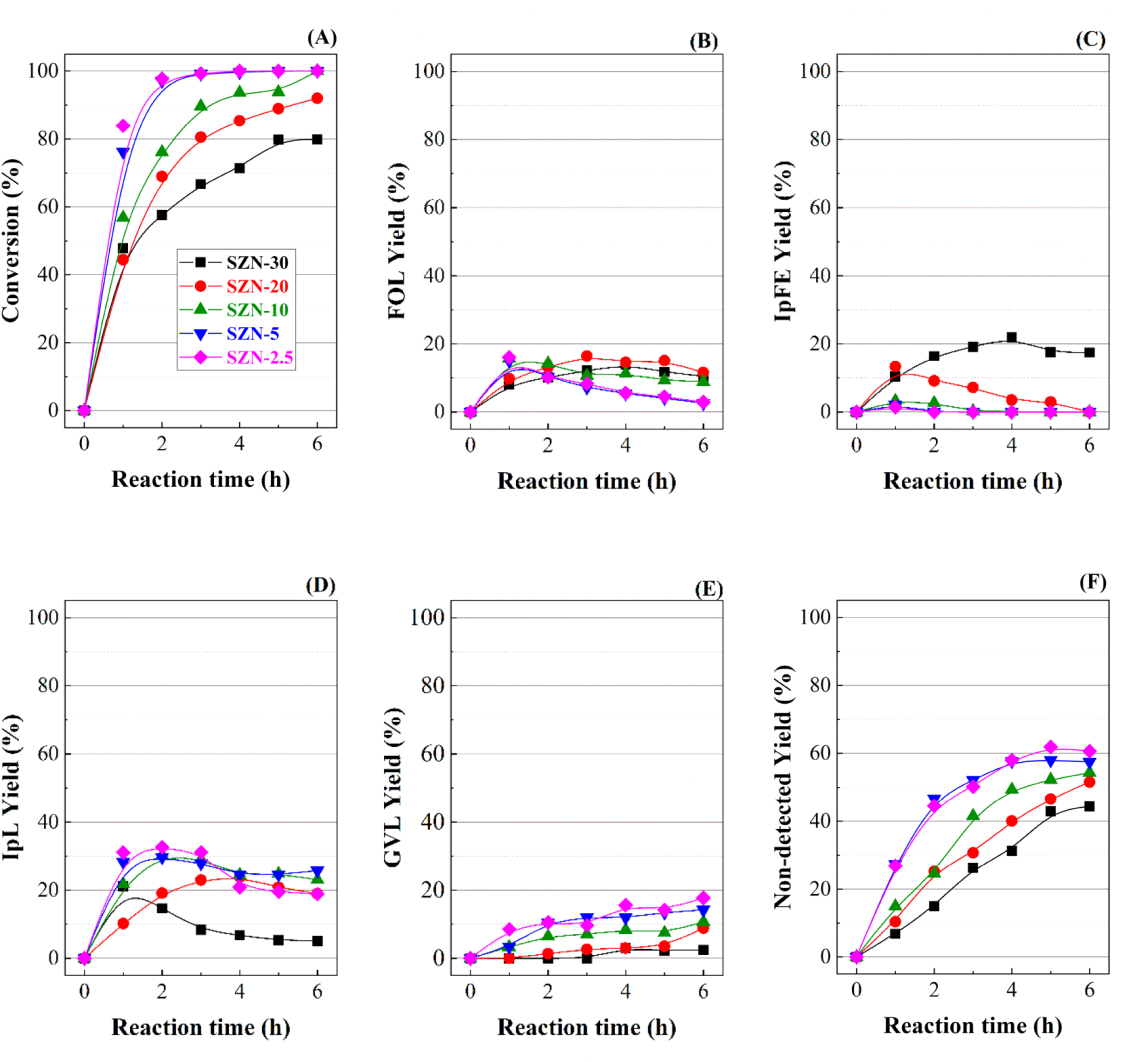
(A) FUR conversion, (B) FOL yield, (C) IpFE yield, (D) IpL yield, (E) GVL yield, and (F) non-detected product yield, in the CTH reaction of FUR using SZN-*x* catalysts with different Si/Zr molar ratios (experimental conditions: temperature, 170 °C; i-Pr–OH/FUR molar ratio, 50; 0.1 g of catalyst; FUR/catalyst weight ratio, 1).

Once SZN-*x* catalysts were characterized, these were evaluated in the conversion of FUR into value-added chemicals, with the participation of their Lewis and Brönsted acid sites by consecutive reactions (Scheme 1). To evaluate the catalytic behavior of these catalysts, two temperatures (110 and 170 °C) were selected to carry out the first tests (Figures 9 and 10).

At 110 °C (Figure 9), the FUR conversion increases with both the Zr content of the catalysts and the reaction time (Figure 9A). Thus, the catalyst with the highest Zr loading (SZN-2.5) reaches a FUR conversion of 76% after 6 h of reaction, while the catalyst with the lowest Zr content (SZN-30) only achieves a conversion of 30% under similar experimental conditions. Regarding the selectivity pattern, all the catalysts give rise to a wide range of products, because of the coexistence of Lewis and Brönsted acid sites (Table 2), which would favor cascade reactions as was reported in the literature by previous authors working with Zr-doped zeolites.^31−33^ In any case, FUR conversion increases with the acidity of SZN-*x* catalysts.

All catalysts exhibit a low selectivity toward FOL (Figure 9B), attaining a maximum yield of 7% after 6 h of reaction. This is a valuable product, since it is widely used in the chemical industry for the manufacture of polymers and fine chemicals. In this step, the transformation of FUR to FOL via a CTH process requires Lewis acid sites, being 2-propanol is adsorbed on the unsaturated Zr species (Lewis acid sites). Moreover, the electron-rich oxygen of the carbonyl group of FUR is also coordinated to these Zr sites bonded to isopropoxide species, and through a six-membered intermediate,^11,60^ a hydride of the isopropoxide is transferred to the carbonyl group of FUR, FUR is reduced to FOL, and concomitantly the sacrificing alcohol (2-propanol) is oxidized to acetone.^11^ It has been reported in the literature that 2-propanol and 2-butanol (secondary alcohols) are considered as better hydrogen donors than primary alcohols, such as methanol and ethanol.^60^ In general, primary alcohols give rise to aldehydes after the CTH reaction, which are highly reactive and tend to form polymeric compounds or humins, by self-condensation reactions.^60^ Considering that the FOL yield is very low in all cases and the CTH reaction is the first stage in these consecutive reactions of FUR, it is expected that the catalytic process evolves toward more advanced stages in this one-pot process.

Subsequently, FOL can be etherificated with the sacrificing alcohol to form i-propyl furfuryl ether (IpFE) (Figure 9C). In this step, a proton coming from a Brönsted acid site would attack the O of the hydroxyl group of a FOL molecule, facilitating the etherification with 2-propanol, releasing H_2_O to form IpFE. Lewis acid sites can also promote this reaction by the coordination of Zr sites with FUR to improve its electrophilicity and a subsequent attack of the hydroxyl group of 2-propanol to form IpFE.^33,60^ IpFE is also considered as valuable product due to alkyl furfuryl ethers have been proposed as gasoline additive, since they increase the octane number.^39,65^ From Figure 9C, it can be observed how IpFE yield increases along the reaction time for those catalysts with lower Zr content (SZN-30 and SZN-20), reaching a maximum value close to 30% after 6 h of reaction. However, the catalysts with higher Zr loading only show high yield toward IpFE at shorter reaction times, achieving a maximum value of 31% after 3 h for SZN-10 catalyst, which decreases (9–18%) for longer reaction times (6 h). This would mean that IpFE is transformed into other reaction products at longer reaction time.

Both FOL and IpFE can undergo a rehydration reaction, which causes the opening of the furan ring, giving rise to levulinic acid (LA) or alkyl levulinate, in the presence of Brönsted acid sites.^33^ However, Brönsted acid sites can also promote a side reaction, which can lead to soluble and insoluble polymers,^33^ although SZN-*x* catalysts possess a low proportion of Brönsted acid sites in comparison to their total acidity (Table 2). In this step, IpFE, or FOL, is protonated by Brönsted acid sites, releasing 2-propanol or H_2_O, respectively. The obtained intermediate is attacked by the alcohol and H_2_O causing the opening of the furan ring to form i-propyl levulinate (IpL) (Figure 9D).^60^ This chemical can be used as solvent,^66^ fuel additive,^67^ flavoring, or plasticizers.^39^ The catalytic data indicate that the catalysts with lower Zr contents (SZN-30 and SZN-20) barely favor the formation of IpL at 110 °C. However, the catalysts with higher Zr content show a progressive increase in IpL yield along the reaction time, achieving a maximum IpL yield of 39% for SZN-2.5, after 6 h of reaction.

On the other hand, the formation of non-detected products also increases along the reaction time, being more pronounced for those catalysts with higher Zr content (Figure 9E). Thus, the yield toward non-detected products ranges from 10% for SZN-30, SZN-20, and SZN-10 catalysts to 25% for SZN-2.5. The presence of non-detected products can be ascribed to the high reactivity of FUR and FOL, which can lead to uncontrolled polymerization reactions, being deposited on the catalyst surface, as was previously observed in other works.^17,52^

The catalytic behavior of the SZN-*x* catalysts was also evaluated at 170 °C (Figure 10). In all cases, FUR conversion increases faster, even at shorter reaction times (Figure 10A). This conversion increase is more pronounced in the case of the catalysts with higher Zr content, since SZN-2.5 and SZN-5 catalyst reach almost full conversion after 2 h of reaction at 170 °C. In the case of catalysts with lower Zr content, the increase in FUR conversion is more gradual, attaining a value of 80% for SiZr-30, after 6 h of reaction. Regarding the products obtained, FOL yield increases after the first hours of reaction (Figure 10B), but it is striking that the catalysts with lower Zr content show a slower, but progressive growth of the FOL yield, whereas the catalysts with higher Zr content reach a higher FOL yield at shorter reaction time (about 15% after 1–2 h of reaction). From this point, FOL can lead to other products, or deactivate due to polymerization processes. The comparison of the FOL yield between 110 and 170 °C reveals that the formation of FOL is enhanced at higher temperature, in particular for higher Zr content, although these values decay after 1–2 h of reaction.

In the case of IpFE (Figure 10C), the formation of alkyl furfuryl ether is only observed with those catalysts with lower Zr content, reaching a maximum IpFE yield close to 20% for SZN-2.5 from 3 h of reaction, while those catalysts with a higher Zr content display very low IpFE yields. These data differ from those obtained at 110 °C, since the catalysts with higher Zr content showed a low IpFE yield.

IpL is the most abundant product detected at 170 °C, mainly for the highest Zr content (SZN-2.5), which achieves a maximum yield of 33% at 2 h (Figure 10D). Therefore, it can also be inferred that the transformation of FUR does not go much forward with the catalysts with lower Zr content, as deduced from the IpL yields.

The use of higher reaction temperature also evidences the presence of GVL (Figure 10E). This product requires Lewis acid sites for the reduction of the ketone group of IpL to form i-propyl 4-hydroxypentanone, through a CTH reaction, with 2-propanol as the sacrificing alcohol. This reaction displays a less favorable thermodynamic equilibrium, since both i-propyl 4-hydroxypentanone and 2-propanol are secondary alcohols.^33^ However, i-propyl 4-hydroxypentanone is not observed, so it must react rapidly through a intramolecular lactonization reaction, which is catalyzed by Brönsted or Lewis acid sites.^33^ GVL is also considered a valuable product, since it is a green solvent. In addition, its herbal odor also favors its use in the fragrance and flavor industries.^68^ The catalytic results indicate that the use of catalysts with higher Zr content favor the formation of GVL, reaching a maximum value of 18% with SZN-2.5, after 6 h of reaction.

Finally, the amount of non-detected products increases when the reaction temperature rises, which could be explained by the formation of carbonaceous deposits (Figure 10F). Thus, the catalysts with higher Zr content (SZN-5 and SZN-2.5) give rise to non-detected product yields between 61 and 67%.

**Figure 11:**
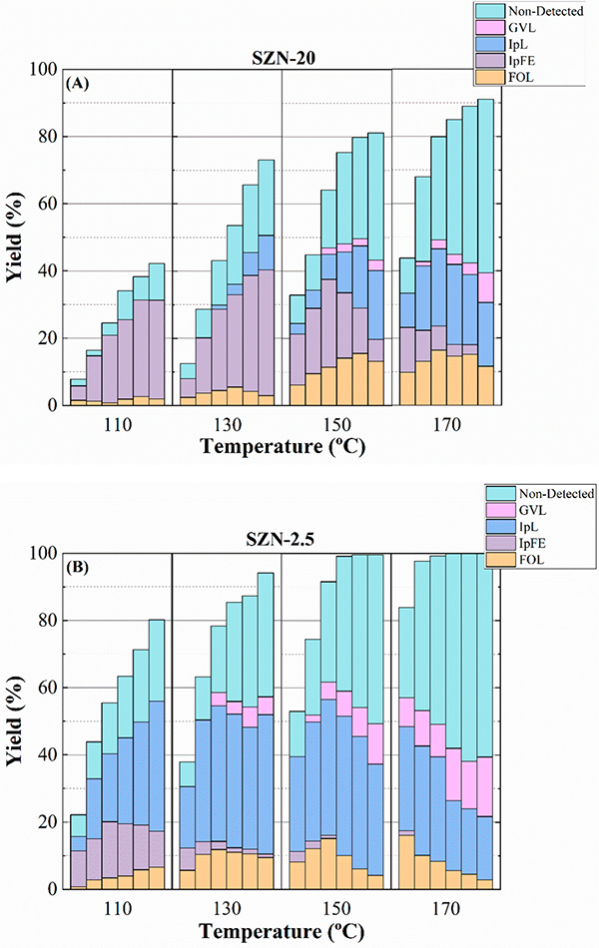
Yield patterns in the CTH reaction of FUR using (A) SZN-20 and (B) SZN-2.5 catalysts (experimental conditions: temperature, 110–170 °C; reaction time, 2h; i-Pr–OH/FUR molar ratio, 50; 0.1 g of catalyst; FUR/catalyst weight ratio, 1).

To evaluate the effect of the reaction temperature on the catalytic activity, two catalysts with different acidity (SZN-20 and SZN-2.5) were selected (Figure 11). Among them, the catalyst with lower acidity (SZN-20) requires higher temperature to reach high conversion values (Figure 11A), being IpFE the main product at lower temperature (110 °C). These data are in agreement with kinetic studies, since the etherification is thermodynamically favored at lower temperature.^61^ The catalytic tests at higher temperature show a decrease in the IpFE yield, which is accompanied by a slight rise of the FOL yield. However, the most relevant data are related to an increase in IpL yield, as well as the formation of GVL. In addition, the increase in the temperature also favors the formation of non-detected products. Therefore, a high reaction temperature promotes consecutive reactions, for which IpL is the main product.

A catalyst with a higher acidity, such as SZN-2.5 (Figure 11B), attains higher FUR conversion values at shorter reaction times, and the coupling of different catalytic steps in one-pot processes is favored. Thus, when the reaction takes place at 110 °C, a clear increase in IpL yield is attained compared to the catalyst with lower acidity (SZN-20). This fact can be ascribed to the higher proportion of Lewis acid sites, which favor the formation of FOL and IpFE. These products evolve to IpL in the presence of Brönsted acid sites, although it must be considered that the proportion of Brönsted acid sites is similar in both cases. The increase in the temperature causes a drastic decrease in IpFE yield, while IpL and GVL yields improve, so it can be thought that a higher proportion of acid sites can favor consecutive reactions, even at shorter times and lower temperatures. Thus, it has been previously proposed that the transformation IpL → GVL requires high temperature to accomplish the lactonization step.^60,61^ Finally, a higher proportion of acid sites also favors side reactions, also increasing the amount of non-detected products.

**Figure 12:**
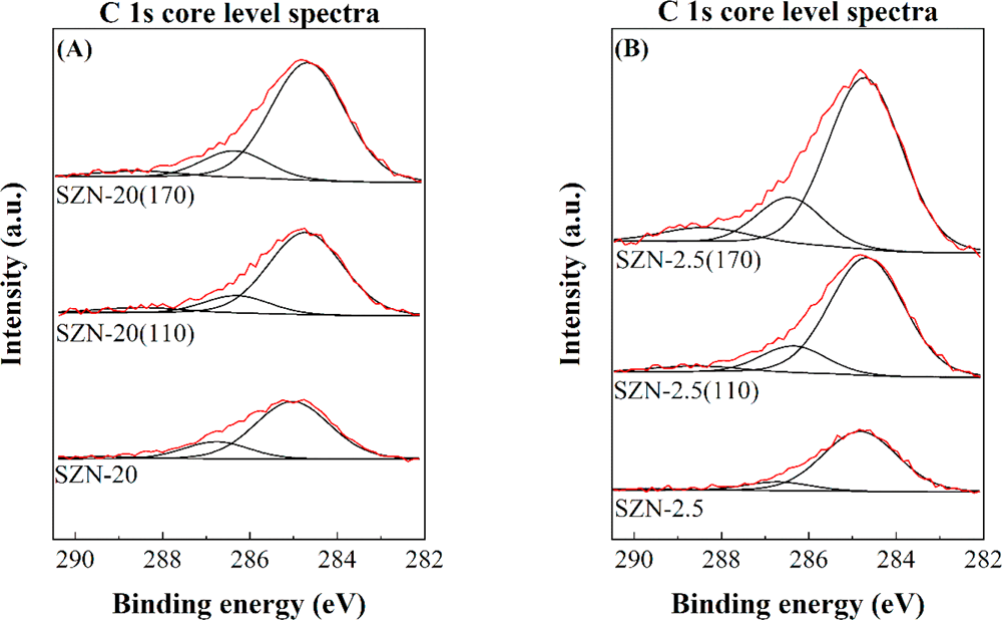
C 1s core level spectra of (A) SZN-20 and (B) SZN-2.5 catalysts, after the reaction (experimental conditions: temperature, 110–170 °C; reaction time, 2 h; i-Pr–OH/FUR molar ratio, 50; 0.1 g of catalyst; FUR/catalyst weight ratio, 1).

**Figure 13:**
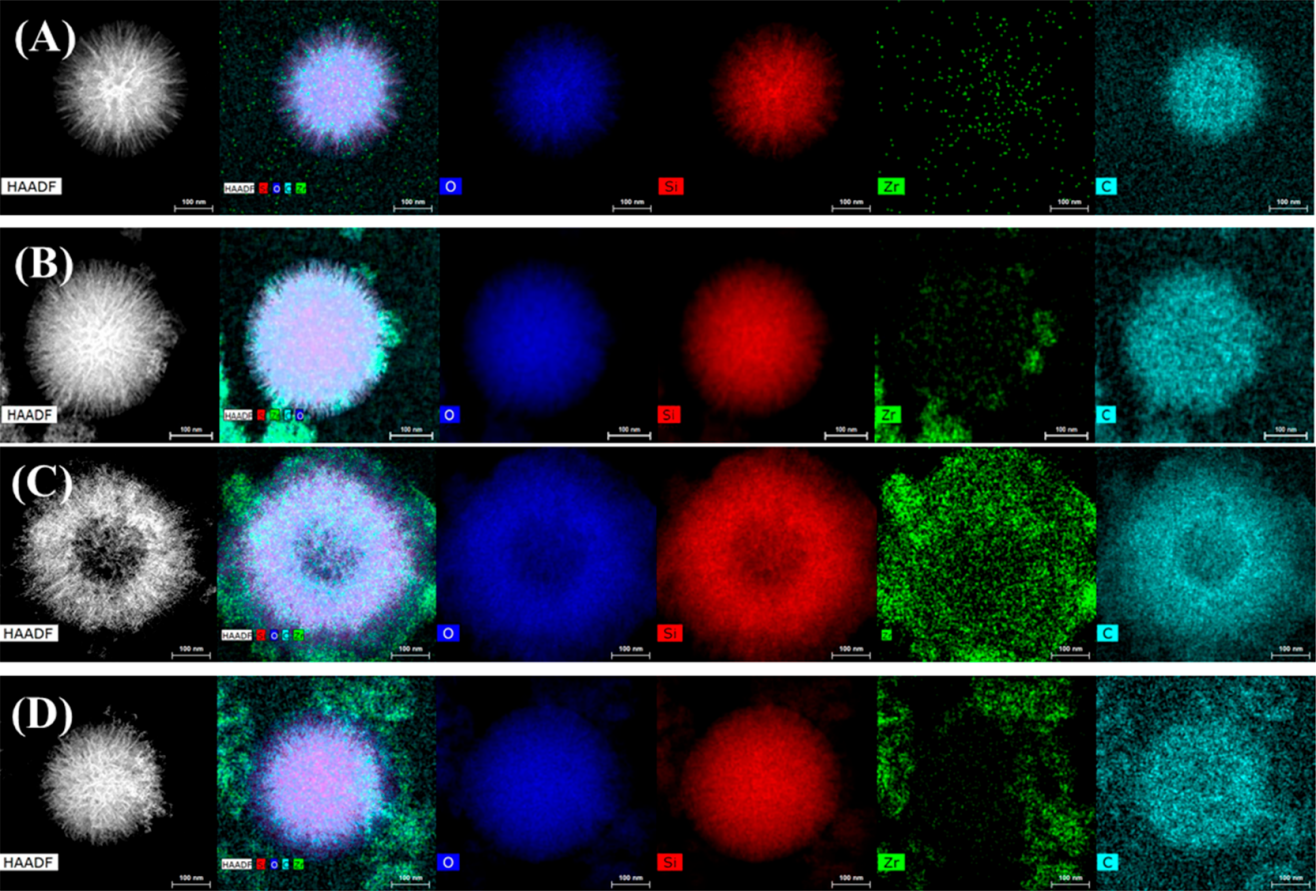
TEM micrographs of (A) SZN-20, (B) SZN-2.5, (C) SZN-5, and (D) SZN-5-O after the reaction (experimental conditions: temperature, 170 °C; reaction time, 2 h; i-Pr–OH/FUR molar ratio, 50; 0.1 g of catalyst; FUR/catalyst weight ratio, 1). Scale: 100 nm.

After the reactions at 110 and 170 °C, the catalysts were recovered to be characterized by XPS and TEM. The O 1s, Si 2p, and Zr 3d core level spectra show the same contributions observed for fresh catalysts, although their surface concentration diminishes due to the existence of carbonaceous species on the catalyst surface (Figure 12 and Table 4). In the C 1s core level spectra, the catalyst with a lower Zr content (SZN-20), which also has a lower proportion of acid sites, shows an increase in the surface carbon content lower than the catalyst with a higher acidity (SZN-2.5). In addition, the use of higher reaction temperature also implies an increase in the C content on the catalyst surface. These data are in agreement with the higher amount of non-detected products observed at 170 °C. The surface Si/Zr molar ratio in the used catalysts increases, so a preferential location of the carbonaceous matter on the Zr species is expected. The morphology of the used catalysts, after 2 h at 170 °C, was also studied by TEM (Figure 13). Zr species, located in the spherical nanoparticles of SZN-20, can be distinguished; however, the catalyst with a higher Zr content maintains the partial segregation of ZrO_2_. In addition, these micrographs reveal the presence of carbon in the SZN-*x* nanoparticles, confirming the deposition of carbonaceous species.

**Table 4:** XPS Data of SZN-20 and SZN-2.5 Catalysts, Fresh and Used^a^

	Atomic Concentration,%/(Binding Energy, eV)				
catalyst	C 1s	O 1s	Si 2p	Zr 3d	Si/Zr atomic ratio
SZN-20	3.51/(284.8)	66.89/(532.9)	28.29/(103.7)	0.16/(182.9)	176.81
	1.04/(286.4)				
	0.13/(289.4)				
SZN-20–110	5.83/(284.7)	64.64/(532.7)	27.73/(103.4)	0.14/(182.7)	198.07
	1.26/(286.3)				
	0.37/(288.6)				
SZN-20–170	8.47/(284.8)	62.61/(532.7)	26.47/(103.4)	0.14/(182.6)	189.07
	1.94/(286.5)				
	0.44/(288.7)				
SZN-2.5	3.73/(284.8)	67.5	19.89/(103.0)	8.46/(182.8)	2.35
	0.54/(286.7)	53.34/(532.3)			
	0.10/(289.6)	14.15/(530.5)			
SZN-2.5–110	13.88/(284.8)	45.86/(532.1)	16.21/(103.2)	6.67/(182.7)	2.43
	3.79/(286.5)	12.41/(530.4)			
	1.19/(288.5)				
SZN-2.5–170	23.4	42.03/(532.5)	15.49/(103.2)	6.17/(182.7)	2.51
	17.63/(284.8)				
	4.39/(286.5)	12.90/(530.4)			
	1.38/(288.5)				

a Experimental conditions: temperature, 110-170 °C; reaction time, 2 h; i-Pr–OH/FUR molar ratio, 50; 0.1 g of catalyst; FUR/catalyst weight ratio, 1.

**Figure 14:**
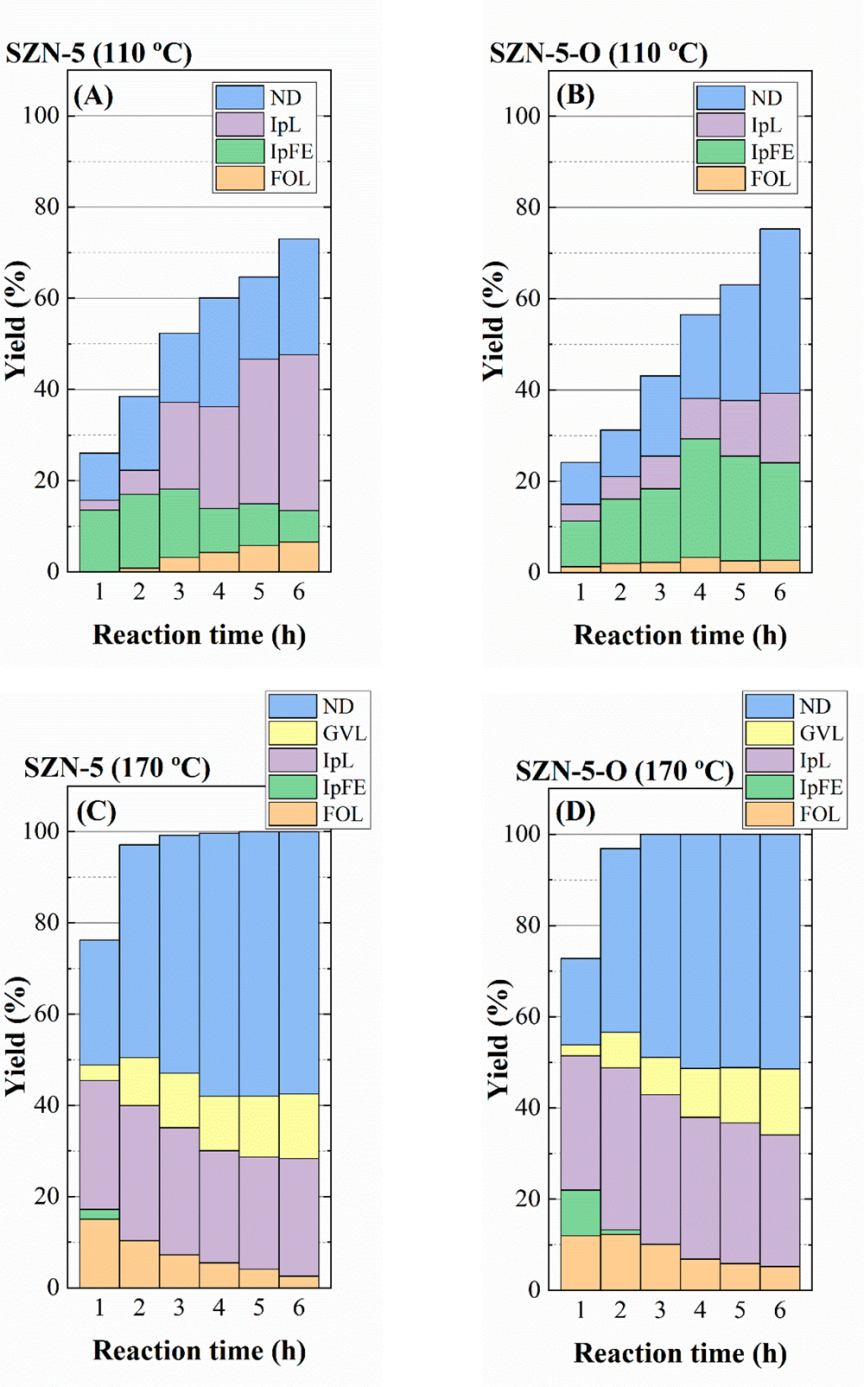
Yield patterns in the CTH reaction of FUR using SZN-5 and SZN-5-O catalysts (experimental conditions: temperature, 110–170 °C; reaction time, 2 h; i-Pr–OH/FUR molar ratio, 50; 0.1 g of catalyst; FUR/catalyst weight ratio, 1).

The yield patterns of catalysts synthesized using zirconium oxychloride (SZN-5-O) or zirconium alkoxide (SZN-5) only display slight differences between them (Figure 14). At 110 °C, similar FUR conversion values are attained (Figure 14A,B), although SZN-5-O gives rise to a higher IpFE yield, whereas SZN-5 allows the reaction to go forward to form IpL, a more advanced stage in the catalytic conversion of FUR, according to Scheme 1, but under mild reaction temperature. It must be taken into account that the concentration of Brönsted acid sites is higher in SZN-5 (Table 2), thus favoring the conversion of IpFE into IpL (Scheme 1). At higher reaction temperature (170 °C) (Figure 14C,D), all the catalytic stages go forward, and SZN-5-O attains higher IpL and GVL yields, whereas SZN-5 shows a larger proportion of nondetected (ND) products, which can be explained by its higher concentration of Brönsted acid sites. On the other hand, it must be considered that the formation of FOL can also lead to nondetected products, since it tends to polymerize with itself or with FUR molecules.

**Figure 15:**
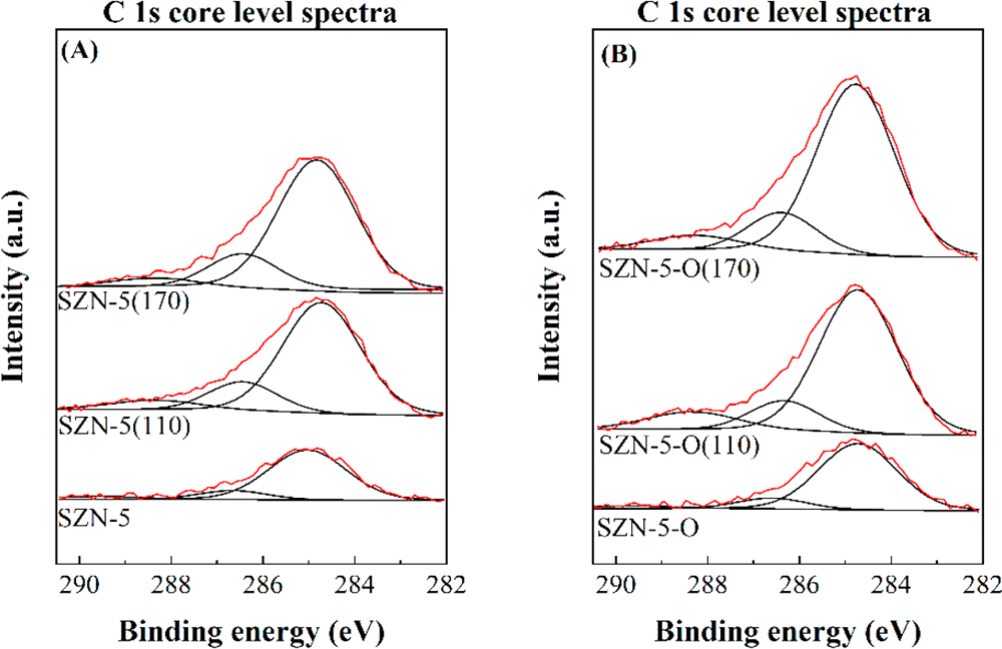
C 1s core level spectra of (A) SZN-5 and (B) SZN-5-O catalysts, after the reaction (experimental conditions: temperature, 110–170 °C; reaction time, 2 h; i-Pr–OH/FUR molar ratio, 50; 0.1 g of catalyst; FUR/catalyst weight ratio, 1).

The analysis of used SZN-5 and SZN-5-O by XPS does not reveal important modifications in the C 1s, O 1s, Si 2p and Zr 3d core level spectra (Table 5). However, it is also noticeable the increase in the surface carbon content, after the reaction, mainly in the case of SZN-5-O (Figure 15), which displays a higher surface Zr content. This is accompanied by an increase in the superficial Si/Zr molar ratio due to the preferential deposition of carbonaceous species on the Zr species, previously observed for other SZN-*x* catalysts. The presence of carbon in the used catalyst was confirmed by TEM (Figure 13).

**Table 5:** XPS Data of SZN-5 and SZN-5-O Catalysts, Fresh and Used^a^

	atomic concentration,%/(binding energy, eV)				
catalyst	C 1s	O 1s	Si 2p	Zr 3d	Si/Zr atomic ratio
SZN-5	3.49/(284.8)	59.39/(532.7)	23.70/(103.2)	4.33/(183.0)	5.47
	0.63/(286.5)	7.85/(530.5)			
	0.21/(289.4)				
SZN-5–110	14.67	54.43/(532.5)	20.91/(103.1)	3.59/(182.6)	5.82
	10.86/(284.8)	6.39/(530.6)			
	2.86/(286.5)				
	0.95/(288.4)				
SZN-5–170	12.95/(284.8)	58.44	20.65/(103.2)	3.62/(182.7)	5.70
	3.37/(286.4)	52.27/(532.6)			
	0.97/(288.3)	6.17/(530.7)			
SZN-5-O	3.88/(284.8)	58.66/(532.4)	21.04/(103.1)	5.56/(182.9)	3.78
	0.64/(286.6)	5.08/(530.5)			
	0.16				
SZN-5-O-110	15.02/(284.8)	49.12/(532.3)	17.76/(102.8)	4.18/(182.6)	4.24
	3.14/(286.4)	8.89/(530.5)			
	1.88/(288.4)				
SZN-5-O-170	16.90/(284.8)	55.86	17.79/(103.0)	4.11/(182.7)	4.33
	3.87/(286.4)	48.67/(532.3)			
	1.47/(288.3)	7.19/(530.5)			

a Experimental conditions: temperature, 110–170 °C; reaction time, 2 h; i-Pr–OH/FUR molar ratio, 50; 0.1 g of catalyst; FUR/catalyst weight ratio, 1.

**Figure 16:**
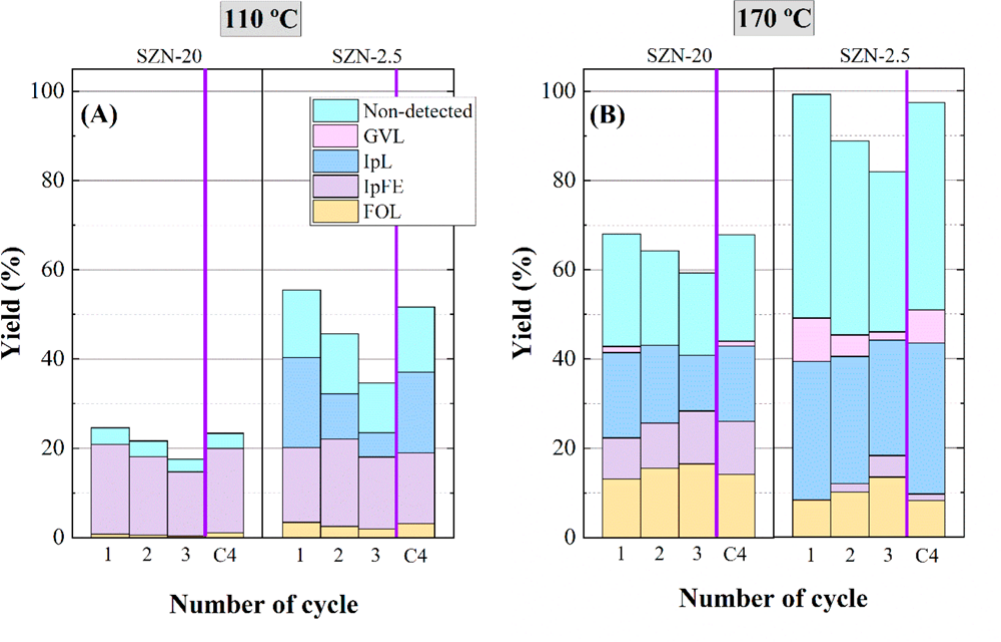
Yield patterns in the CTH reaction of FUR using (A) SZN-20 and (B) SZN-2.5 catalysts in the reusing study (experimental conditions: temperature, 110–170 °C; reaction time, 2 h; i-Pr–OH/FUR molar ratio, 50; 0.1 g of catalyst; FUR/catalyst weight ratio, 1).

The sustainability of these catalysts was finally evaluated in a reutilization study of SZN-20 and SZN-2.5 during several catalytic cycles. It should be noted that the catalysts were only filtered between consecutive cycles, except in the last cycle (C4), where the catalyst was also calcined at 600 °C to remove the organic species deposited on its surface (Figure 16). The FUR conversion values decrease after each catalytic cycle, and the relationship between total acidity and catalytic activity is confirmed. However, the catalyst with a higher amount of acid sites (SZN-2.5) is also more prone to undergo deactivation, causing a stronger decay of conversion values, due to a higher amount of carbonaceous deposits. In addition, the decrease in FUR conversion is more pronounced at high reaction temperature (170 °C), at which deactivation is more important also due to these carbonaceous deposits. In any case, the calcination of the SZN-2.5 and SZN-20 catalysts at 600 °C allows the recovery of almost all the initial catalytic activity, in terms of both FUR conversion and yield pattern. The slight difference between cycle-1 (C1) and cycle-4 (C4) could be ascribed to the loss of acid sites, because of particle sintering in the regeneration step. Concerning the yield pattern, a change in the ratio of obtained products can be observed along the catalytic cycles. The catalytic activity, in general, decreases due to the presence of carbonaceous deposits, since the covering of Brönsted and Lewis acid sites limits the advance of the transformation of FUR. Thus, the main reaction products affected are IpL and GVL. At 170 °C, a concomitant increase in the formation of FOL and IpFE is observed, together with a progressive decrease in the amount of nondetected products.

**Figure 17:**
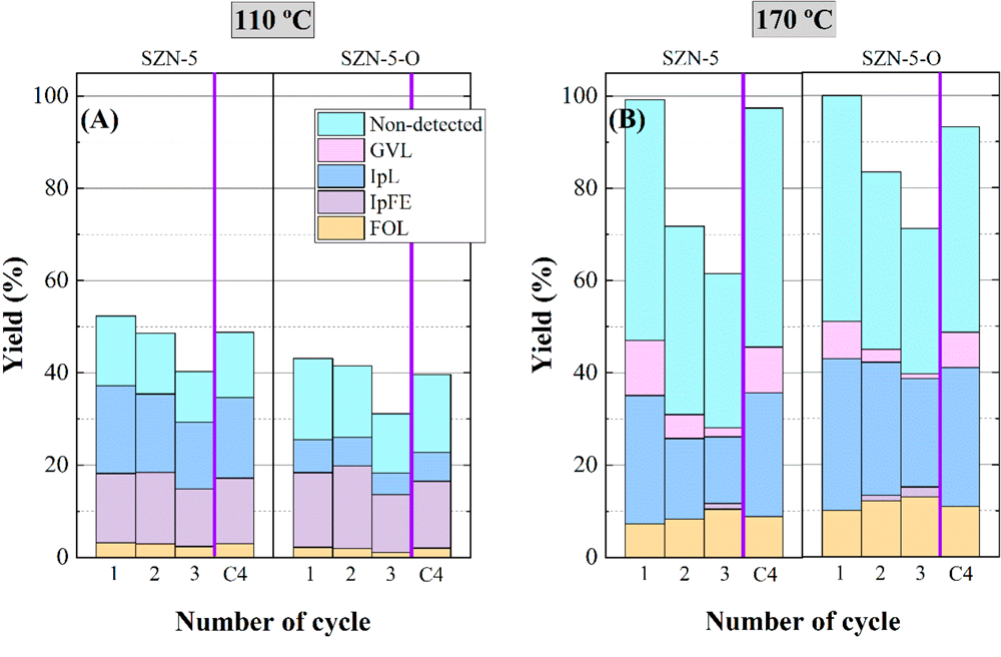
Yield patterns in the CTH reaction of FUR using (A) SZN-5 and (B) SZN-5-O catalysts in the reusing study (experimental conditions: temperature, 110–170 °C; reaction time, 2 h; i-Pr–OH/FUR molar ratio, 50; 0.1 g of catalyst; FUR/catalyst weight ratio, 1).

A similar trend is found in the reutilization study of SZN-5 and SZN-5-O catalysts (Figure 17). FUR conversion of both catalysts diminishes with the number of cycles due to the deposition of carbonaceous species. At 170 °C, the decay of FUR conversion is accompanied by a modification of the proportion of the obtained products, since both GVL and IpL decrease, while FOL and IpFE are favored.

The reaction liquid, after the first catalytic cycle, was collected to evaluate the possible catalyst leaching. The elemental analysis, determined by ICP, showed that the leaching of Si and Zr can be considered negligible, confirming that the catalytic process is carried out under heterogeneous conditions. These data agree with those previously reported, where the leaching was hardly observed.^29,31^

## 4 Conclusions

Silica nanospheres have been modified by incorporating different amounts of ZrO_2_, using two synthetic strategies. The characterization data reveal that the loading of a small amount of Zr in the synthesis step favors their incorporation in the silica nanospheres. However, higher proportions of Zr give rise to a partial segregation of ZrO_2_, although the particles formed are not detected by XRD due to their small size. As the Zr content increases, the textural properties worsen, but the number of acid centers increases due to the generation of a higher proportion of Lewis acid sites, although a small proportion of Brönsted was also observed.

The presence of appropriate textural properties, as well as the coexistence of Brönsted and Lewis acid centers, have prompted its use as catalysts in the conversion of furfural, through one-pot reactions, to several high value-added chemicals. The catalytic results showed that the increase in the acidity improves FUR conversion, obtaining IpFE and IpL at lower temperature (110 °C) and IpL and GVL at higher temperature (170 °C). In addition, it is also noteworthy that the proportion of nondetected products increases for a lower Si/Zr molar ratio and higher temperature, due to the high reactivity of FUR and FOL, which tend to polymerize in the presence of acid sites. This leads to the formation of carbonaceous deposits on the surface of the SZN nanoparticles, causing a progressive deactivation after several catalytic cycles. However, the catalysts can be easily regenerated by calcination to remove carbonaceous deposits, obtaining FUR conversion and a selectivity pattern similar to those of fresh catalysts.

The next challenge in the one-pot reaction of FUR will be to adjust the concentration of acid sites, as well as the modulation of the amount of Lewis and Brönsted acid sites, to decrease undetected products. Moreover, it is necessary to optimize the synthesis method to be able to incorporate large amounts of ZrO_2_ into the SiO_2_ nanospheres.
